# Does Attention‐Deficit/Hyperactivity Disorder Predominant Presentation Matter? Examining Functional and Symptom Changes After Cognitive Behavioural Therapy

**DOI:** 10.1002/cpp.70271

**Published:** 2026-04-17

**Authors:** Juan Jesús Crespín, Montse Corrales, Vanesa Richarte, Gemma Parramon‐Puig, Paula Querol, Ferran Mestres, Carolina Ramos‐Sayalero, Maria Boix, Carla Torrent, Christian Fadeuilhe, Josep Antoni Ramos‐Quiroga, Silvia Amoretti

**Affiliations:** ^1^ Department of Mental Health Hospital Universitari Vall d'Hebron Barcelona Catalonia Spain; ^2^ Group of Psychiatry, Mental Health and Addictions, Vall d'Hebron Research Institute (VHIR) Barcelona Spain; ^3^ Department of Psychiatry and Forensic Medicine Universitat Autònoma de Barcelona Barcelona Spain; ^4^ Biomedical Research Networking Center for Mental Health Network (CIBERSAM), ISCIII Barcelona Spain; ^5^ Bipolar and Depressive Disorders Unit, Hospital Clínic de Barcelona; Institut de Neurociències (UBNeuro); Fundació Clínic‐Institut d'Investigacions Biomèdiques August Pi I Sunyer (IDIBAPS) Barcelona Spain; ^6^ Departament de Medicina, Facultat de Medicina i Ciències de la Salut Universitat de Barcelona (UB) Barcelona Spain

**Keywords:** ADHD, ADHD presentations, cognitive behavioural therapy, randomized controlled trial, symptom severity, treatment response

## Abstract

**Background:**

Cognitive behavioural therapy (CBT) is an effective intervention for adults with attention‐deficit/hyperactivity disorder (ADHD). While both brief (6‐session) and standard (12‐session) formats show efficacy, it remains unclear whether treatment response differs between distinct ADHD clinical presentations.

**Methods:**

This secondary analysis of a randomized trial included 80 adults with ADHD (57.5% male; mean age = 41.26 ± 9.32 years) allocated to brief or standard CBT. Clinical and functional outcomes were assessed at baseline, posttreatment, as well as 3‐ and 6‐month follow‐ups. Linear mixed‐effects models and the Reliable Change Index (RCI) evaluated interactions between time, treatment format and ADHD presentation (inattentive vs. combined).

**Results:**

CBT yielded significant improvements across all domains, with 94.5% of participants achieving reliable symptomatic improvement. A significant time × presentation interaction (*p* = 0.001, *ηp* ^2^ = 0.135) revealed a steeper core symptom reduction in the combined group, which survived sensitivity analyses controlling for baseline severity. Regarding format, the brief version produced greater observer‐rated symptom improvement, whereas the standard format yielded greater long‐term functional gains (*p* = 0.009, *ηp* ^2^ = 0.061). Improvements were sustained at the 6‐month follow‐up. No robust three‐way interactions emerged.

**Conclusions:**

CBT is highly effective and durable across ADHD presentations and formats. Although the combined presentation exhibited a more pronounced reduction in core symptoms, this differential trajectory represents a promising clinical trend requiring cautious interpretation. While brief formats efficiently address core symptoms, standard programs may better optimize long‐term functional recovery.

## Summary

- CBT produces clinically significant and durable improvements in core ADHD symptoms, daily functioning and comorbid emotional distress in adults, with approximately 95% of participants achieving reliable symptomatic improvement.
- Adults with the combined presentation show a steeper trajectory of improvement in core symptoms following CBT, reflecting a genuine clinical response even when accounting for their greater baseline severity
- Both brief (6‐session) and standard (12‐session) formats are broadly effective across clinical presentations; however, the standard format may offer additional long‐term benefits for functional recovery.
- Clinicians should consider ADHD presentations when tailoring treatment intensity, while recognizing that these subtype‐specific differential trajectories represent promising clinical trends that require replication.

## 1 Introduction

Attention‐deficit/hyperactivity disorder (ADHD) is a prevalent neurodevelopmental condition that typically begins in childhood and, in most cases, persists into adulthood (American Psychiatric Association 2022). Although traditionally considered a childhood disorder, evidence indicates that approximately two‐thirds of affected children continue to meet diagnostic criteria in adult life (Caye et al. 2016). The estimated global prevalence of ADHD in adults is approximately 3.3%; however, this figure varies substantially by methodology, with register‐based studies reporting rates as low as 0.2%, likely reflecting persistent underdiagnosis and undertreatment in this population (Popit et al. 2024). In adulthood, ADHD substantially impairs academic performance, occupational functioning and interpersonal relationships (Holst and Thorell 2020).

ADHD is a heterogeneous condition with regard to its aetiology, symptom profile and functional impact (Nigg et al. 2010; Faraone et al. 2024). To reflect this heterogeneity, the DSM‐5‐TR specifies three clinical presentations: predominantly inattentive, predominantly hyperactive–impulsive and combined (American Psychiatric Association 2022). Symptom expression evolves throughout the lifespan, with inattentive features often becoming more prominent in adulthood, while hyperactivity tends to decline (Das et al. 2012). In one adult clinical sample, 18.3% of individuals were classified as predominantly inattentive, 8.3% as predominantly hyperactive/impulsive, and 70% as combined (Salvi et al. 2019). The hyperactive/impulsive presentation was more common among women, the inattentive presentation more frequently observed in men, and individuals with the hyperactive/impulsive profile reported lower quality of life and higher rates of anxiety disorders (Salvi et al. 2019). Because this presentation is rare in adult samples—largely due to the internalization or remission of overt symptoms over time (Das et al. 2012)—it is consistently underrepresented in clinical trials. The present study therefore focuses exclusively on the inattentive and combined presentations, in line with most adult ADHD research.

A central debate concerns whether ADHD presentations represent a severity continuum or distinct clinical entities (Wu et al. 2022). The combined presentation is consistently associated with greater overall symptom severity, higher rates of externalizing comorbidities, such as conduct disorder and substance use disorder, and elevated psychiatric burden, including lifetime bipolar disorder and psychosis (Wilens et al. 2003, 2009). Developmentally, this profile often follows a ‘persistent‐high’ trajectory from childhood, linked to reactive aggression and poorer interpersonal outcomes (Murray et al. 2020). In contrast, the inattentive presentation is more closely associated with internalizing symptoms such as anxiety and depression and may follow a ‘late‐onset’ trajectory in which functional impairment emerges only when compensatory strategies are no longer sufficient to meet adult demands (Murray et al. 2020; Rostami et al. 2020). Sex further shapes this pattern: men with ADHD show higher rates of alcohol abuse and conduct disorder, whereas women are more likely to present with dysthymia and severe anxiety (Biederman et al. 1994; Wilens et al. 2009).

Given the complexity of ADHD presentations and their associated impairments, current clinical guidelines recommend a multimodal treatment approach for adults, typically combining pharmacological interventions with psychological and psychoeducational strategies (Kooij et al. 2019). While stimulant medications are widely used, cognitive behavioural therapy (CBT) has emerged as an effective nonpharmacological intervention for adults with ADHD, designed to help individuals develop personalized coping strategies, restructure cognitive distortions and promote adaptive behavioural change (Safren et al. 2005; Knouse and Safren 2010; Young et al. 2020; Gonda et al. 2024). In this population, CBT specifically targets executive dysfunction, emotional dysregulation and maladaptive coping mechanisms and also addresses comorbid symptoms such as anxiety, depression and low self‐esteem (Mongia and Hechtman 2012; Safren et al. 2005). Numerous meta‐analyses and systematic reviews have confirmed that CBT effectively reduces core ADHD symptoms—including inattention, hyperactivity and impulsivity—while also improving executive function, emotional regulation and overall functioning (Liu et al. 2023; Matsumoto et al. 2024; Nimmo‐Smith et al. 2020; Yang et al. 2025). When combined with pharmacotherapy, CBT yields greater improvements in ADHD symptoms and emotional outcomes than medication alone, particularly in the short term (Knouse et al. 2017; Liu et al. 2023; Nimmo‐Smith et al. 2020; Yang et al. 2025). Despite its demonstrated efficacy, CBT remains underutilized in clinical practice because of its duration and resource demands. To address this barrier, recent research has investigated more time‐efficient formats. For instance, a randomized controlled trial by Corrales et al. (2024) found that a six‐session CBT program yielded outcomes comparable to the standard 12‐session format, with sustained improvements at 3‐ and 6‐month follow‐ups. Nevertheless, it remains unclear whether treatment format interacts differentially with specific ADHD clinical profiles. Theoretically, the greater emotional dysregulation and behavioural complexity associated with the combined presentation may require the extended practice and consolidation afforded by the 12‐session format, which incorporates advanced modules for cognitive restructuring and emotional control (Knouse and Safren 2010; Safren et al. 2005). Conversely, individuals with a predominantly inattentive profile may respond equally well to a streamlined six‐session approach focused on core organizational and time‐management strategies (Solanto et al. 2010).

The clinical and functional heterogeneity across ADHD presentations suggests that treatment response—particularly to CBT—may differ according to the predominant clinical profile. This differential response may be explained by the modular structure of evidence‐based CBT protocols (Safren et al. 2005). Because the combined presentation is characterized by higher overall symptom severity, greater emotional dysregulation and more pronounced impulsivity (Rostami et al. 2020; Wilens et al. 2009), CBT components targeting emotional regulation and impulsivity management may be differentially effective for this subgroup (Liu et al. 2023). In contrast, for individuals with the predominantly inattentive presentation, modules addressing attentional deficits, organizational skills and executive dysfunction are likely more central to clinical progress (Solanto et al. 2010). Neurobiological models further suggest that ADHD involves impaired top‐down prefrontal control (Sergeant et al. 2003), a deficit that CBT may partially address by reinforcing executive and compensatory mechanisms (Knouse and Safren 2010). Accordingly, given their higher baseline severity, individuals with the combined presentation may have a greater margin for absolute symptom reduction—commonly described as having ‘more room for improvement’ in clinical trials (Knouse and Safren 2010). Although the overall efficacy of CBT for adult ADHD is well established (Nimmo‐Smith et al. 2020), whether these theoretical and baseline differences translate into differential treatment outcomes remains unclear. We therefore hypothesize that adults with the combined presentation will show greater absolute reductions in clinical symptoms and functional impairment following CBT, driven by their higher baseline severity and the specific relevance of CBT components targeting emotional dysregulation and impulsivity. This study tests this hypothesis, contributing to a more theory‐driven approach to personalized ADHD intervention.

## 2 Method

### 2.1 Sample

This is a secondary analysis of a randomized controlled trial (RCT) conducted at the Adult ADHD Program of the Hospital Universitari Vall d'Hebron (Barcelona, Spain), as described by Corrales et al. (2024). The original study included 81 adults diagnosed with ADHD; however, as only one participant presented with the predominantly hyperactive/impulsive presentation, this individual was excluded from the current analysis, which focuses on the comparative clinical profiles. Minor discrepancies with figures reported in the original trial are attributable to this exclusion. The final sample comprised 80 adults (57.5% male sex at birth; mean age = 41.26 ± 9.32 years). Regarding clinical profile, 30 participants (37.5%) met criteria for the predominantly inattentive presentation and 50 (62.5%) for the combined presentation. Participants were randomly assigned to a brief (6‐session, *n* = 40) or standard (12‐session, *n* = 40) group‐based CBT intervention. The distribution of ADHD presentations did not differ significantly between treatment formats (12‐session: 13 inattentive [32.5%], 27 combined [67.5%]; six‐session: 17 inattentive [42.5%], 23 combined [57.5%]; *χ* ^2^ = 0.853, *p* = 0.356), indicating comparable group composition for the comparative analysis.

Of the final sample, 25 participants were single (31.2%), 43 were married (53.8%), and 12 were separated (15.0%). Regarding educational level, 9 participants had completed primary education (11.3%), 26 secondary education (32.5%), 22 high school or advanced vocational training (27.5%) and 23 university education (28.7%). Concerning employment status, 39 individuals were salaried employees (48.8%), 23 were self‐employed (28.7%), 9 were unemployed or seeking work (11.3%), 5 were students (6.3%), 2 were retired or pensioned (2.5%), and 2 were on medical leave (2.5%). Pharmacological treatment for ADHD included methylphenidate (45.7%), lisdexamfetamine (38.3%) and atomoxetine (16.0%).

Inclusion criteria were as follows: age between 18 and 65 years; an ADHD diagnosis according to DSM‐5‐TR criteria; persistent symptoms indexed by scores ≥ 24 on the ADHD Rating Scale (ADHD‐RS; Richarte et al. 2017) and > 3 on the Clinical Global Impression‐Severity (CGI‐S) scale (Guy 1976); and stable ADHD medication for at least 2 months prior to and throughout the intervention. Exclusion criteria included IQ below 85; a diagnosis of autism spectrum disorder, bipolar disorder, schizophrenia or other psychotic disorder; substance use disorder; or personality disorder according to DSM‐5‐TR. Individuals engaged in other psychological treatments or unable to attend sessions consistently were also excluded.

Randomization was conducted using Research Randomizer software (Version 4; Urbaniak and Plous 2013) by an independent researcher not otherwise involved in the project, who was solely responsible for group allocation. This procedure ensured strict concealment from clinical evaluators and therapists until the intervention commenced. Ethical approval was obtained from the hospital's Clinical Research Ethics Committee (Protocol code: PR[AE]249/2016), and the study was registered on ClinicalTrials.gov (NCT04588181). All participants provided written informed consent and received no financial compensation for their participation.

### 2.2 Assessments

Baseline demographic data on age, sex, educational level, relationship status, living arrangements and employment were collected. ADHD diagnosis and predominant presentation (inattentive or combined) were confirmed through structured interviews conducted by experienced clinicians using the Conners' Adult ADHD Diagnostic Interview (CAADID; Ramos‐Quiroga et al. 2012) and the Diagnostic Interview for ADHD in Adults (DIVA 2.0; Ramos‐Quiroga et al. 2019). Childhood symptomatology was retrospectively assessed at baseline using the Spanish abbreviated version of the Wender Utah Rating Scale (WURS; Ward et al. 1993; Rodríguez‐Jiménez et al. 2001).

Outcome measures were organized into three assessment modalities. Clinician‐administered measures included the Spanish version of the ADHD Rating Scale (ADHD‐RS; Richarte et al. 2017) and the CGI‐S scale (Guy 1976), both administered by trained ADHD‐specialized staff who were blinded to participants' treatment allocation at all assessment points. Self‐report measures, completed independently by participants, included the Conners' Adult ADHD Rating Scales–Self‐Report: Long Version (CAARS‐S:L; Conners et al. 1999; Amador‐Campos et al. 2014), the Beck Depression Inventory‐II (BDI‐II; Beck et al. 1996; Sanz et al. 2003), the State–Trait Anxiety Inventory (STAI; Spielberger et al. 1970; Guillén‐Riquelme and Buela‐Casal 2011), the Functioning Assessment Short Test (FAST; Rosa et al. 2007; Rotger et al. 2014), and the Spanish validation of the WHO Disability Assessment Schedule 2.0 (WHODAS 2.0; Ustün et al. 2010; Amoretti et al. 2025). Observer‐rated symptoms were assessed using the Conners' Adult ADHD Rating Scales–Observer Report: Long Version (CAARS‐O:L; Conners et al. 1999; Amador‐Campos et al. 2014), completed by a significant other (e.g., partner, parent or close relative) familiar with the participant's daily behaviour.

Self‐report and observer‐rated measures were completed in paper‐and‐pencil format outside the clinic to capture symptom experience in naturalistic settings. Trained clinical staff reviewed all completed scales during follow‐up sessions to resolve any ambiguities, verify completeness and ensure consistent responding across assessment points.

All assessments were conducted at baseline, postintervention, as well as at 3‐ and 6‐month follow‐ups, by trained ADHD‐specialized staff blinded to treatment allocation at all time points. Internal consistency of multiitem outcome measures was evaluated using Cronbach's alpha at each assessment point (Table 1). The ADHD‐RS showed acceptable internal consistency at baseline (*α* = 0.72), posttreatment (*α* = 0.68) and the 3‐month follow‐up (*α* = 0.69), falling below acceptable thresholds at 6 months (*α* = 0.57). The FAST showed acceptable to good reliability across time points (*α* range = 0.69–0.81), with the highest values at 6 months. The WHODAS 2.0 demonstrated good internal consistency throughout the study (*α* range = 0.80–0.82).

**TABLE 1:** Internal consistency (Cronbach's *α*) of outcome measures across assessment time points.

Scale	Baseline	Post	3‐Month	6‐Month
ADHD‐RS (18 items)	0.724	0.677	0.688	0.572
FAST (24 items)	0.686	0.691	0.705	0.812
WHODAS 2.0 (12 items)	0.810	0.819	0.812	0.804

Abbreviations: ADHD‐RS, Attention Deficit/Hyperactivity Disorder Rating Scale; FAST, Functioning Assessment Short Test; WHODAS 2.0, World Health Organization Disability Assessment Schedule 2.0.

### 2.3 Interventions

The interventions consisted of two group‐based CBT formats: a 12‐session standard protocol and a six‐session abbreviated version, both delivered at the same weekly frequency and session duration (90 min). The standard protocol was based on an established CBT program for adult ADHD (Safren et al. 2005), targeting psychoeducation, organizational strategies, time and task management, attention regulation, emotional self‐regulation, impulsivity control and relapse prevention. The six‐session program, developed by Corrales et al. (2024), offers a condensed adaptation of the standard protocol, maintaining the same weekly schedule and session length while integrating the essential therapeutic elements: psychoeducation, organization and planning, distraction management, cognitive restructuring, impulsivity and emotional regulation, as well as motivation enhancement with relapse prevention. Specifically, Session 1 introduces the program and provides psychoeducation on ADHD, aiming to increase knowledge and motivation while addressing common misconceptions. Session 2 focuses on time management, organization and problem‐solving. Session 3 targets environmental modification to reduce distractions and sustain attention. Session 4 covers the cognitive model of ADHD and teaches cognitive restructuring techniques to modify negative automatic thoughts. Session 5 addresses impulsivity and emotional dysregulation, providing strategies for behavioural control and relaxation. Session 6 consolidates all previously learned strategies and focuses on relapse prevention and long‐term maintenance. Full details of the session structure and content are provided in Corrales et al. (2024). Both formats were delivered by the same senior clinical psychologist with extensive specialized training in CBT for ADHD. This single‐therapist design was adopted to control for therapist‐related variability and ensure consistency in the delivery of therapeutic components across conditions. Treatment fidelity was maintained through a strictly manualized protocol (Corrales et al. 2024) and regular clinical supervision throughout the trial.

### 2.4 Statistical Analysis

The original trial was designed and powered to assess overall treatment efficacy; accordingly, the current analyses examining interactions by ADHD presentation are secondary and exploratory. To evaluate statistical power for these specific analyses, a sensitivity power analysis was conducted in G*Power for a repeated‐measures within‐between interaction design (time × group). With *α* = 0.05, *N* = 80 participants, a correlation among repeated measures of 0.50, and a nonsphericity correction of *ε* = 1, the study had adequate power to detect moderate effect sizes (Cohen's *f* = 0.25), with achieved power of 0.968 for the two‐timepoint (pre–post) and 0.998 for the four‐timepoint longitudinal analyses. Smaller effects may nonetheless have remained undetected, particularly at the 6‐month follow‐up due to attrition.

Baseline sociodemographic and clinical characteristics were described using standard descriptive statistics. Normality was assessed via Shapiro–Wilk tests and visual inspection of histograms and Q–Q plots. Given the sample size and the robustness of parametric tests to minor normality violations, Student's *t*‐tests and one‐way ANOVAs were used for between‐group comparisons of continuous variables, while Pearson's *χ* ^2^ tests were applied to categorical data. Effect sizes were estimated using Cohen's *d* for continuous measures and Cramér's *V* for categorical variables. A Bonferroni correction was applied to all baseline comparisons and post hoc pairwise contrasts, setting the adjusted significance threshold at *p* ≤ 0.002.

Longitudinal treatment outcomes and presentation differences were evaluated using linear mixed‐effects regression models incorporating random intercepts for each participant to account for the dependency of repeated measures across the four assessment points (baseline, posttreatment, as well as 3‐ and 6‐month follow‐ups). Time and ADHD presentation served as primary fixed factors; treatment format (6 vs. 12 sessions) was included to control for potential duration effects. The primary focus was the examination of time × presentation interaction effects. Missing data were handled using restricted maximum likelihood (REML) estimation, incorporating all available observations under an intention‐to‐treat framework based on the missing‐at‐random assumption. Effect sizes were estimated as partial *η* ^2^ derived from Type III *F*‐tests, with values of approximately 0.01, 0.0 and 0.14 interpreted as small, moderate and large, respectively.

Clinically meaningful improvement was further examined using the Reliable Change Index (RCI; Jacobson and Truax 1991), calculated as the difference between posttreatment and baseline scores divided by the standard error of the difference (S diff = SD baseline × √[2(1 − *r*)]), where *r* is the reliability coefficient of the instrument. Participants were classified as showing reliable improvement (RCI ≤ −1.96), no reliable change (−1.96 < RCI < 1.96) or reliable deterioration (RCI ≥ 1.96). RCI‐based classifications were computed for all primary outcomes from pretreatment to posttreatment. Sensitivity analyses were conducted using complete‐case data from 6‐month completers. Secondary sensitivity analyses included baseline severity of the corresponding outcome as a subject‐level covariate; in these models, baseline observations were excluded from the longitudinal outcome vector to adjust for initial severity and mitigate regression to the mean. All omnibus fixed‐effects tests from the mixed‐effects models were evaluated at *α* = 0.05. All statistical procedures were performed using SPSS version 26 for Windows.

## 3 Results

### 3.1 Differences in Baseline Characteristics by ADHD Predominant Presentation

Baseline sociodemographic and clinical characteristics by ADHD presentation are presented in Table 2. The predominantly inattentive (*n* = 30) and combined (*n* = 50) groups did not differ significantly on any sociodemographic variable.

**TABLE 2:** Baseline differences between ADHD predominant presentations.

	Predominant inattentive presentation (*n* = 30)	Predominant combined presentation (*n* = 50)	*t*	*p* or *χ* ^2^	Effect size
**Age** (mean ± SD)	38.70 ± 9.86	42.80 ± 8.72	−1.938	0.056	0.440
**Sex** (*males*, %)	19 (63.3)	27 (54.0)	0.668	0.281	0.091
**Educational level**: university, *n* (%)	8 (26.7)	15 (30.0)	6.241	0.100	0.279
**Marital status**: single, *n* (%)	13 (43.3)	12 (24.0)	4.590	0.101	0.240
**Living arrangements**: alone, *n* (%)	0 (0.0)	8 (16.0)	10.845	0.055	0.368
**Employment status**: employed, *n* (%)	17 (56.7)	22 (44.0)	2.520	0.773	0.177
**WURS** (mean ± SD)	48.72 ± 15.08	56.36 ± 15.10	−2.143	**0.035**	0.506
**ADHD‐RS** (mean ± SD)	30.97 ± 4.81	37.02 ± 6.89	−4.614	**< 0.001**	1.019
**CAARS‐S:L** (mean ± SD)					
*Inattention*	69.48 ± 10.25	74.31 ± 9.98	−2.042	**0.045**	0.477
*Hyperactivity*	51.38 ± 10.65	64.45 ± 8.12	−6.105	**< 0.001**	1.380
*Impulsiveness*	59.17 ± 13.49	66.80 ± 12.91	−2.478	**0.015**	0.577
*Self‐concept problems*	60.24 ± 9.37	63.82 ± 12.13	−1.363	0.177	0.330
ADHD Global Index	64.76 ± 9.44	73.00 ± 9.27	−3.768	**< 0.001**	0.880
**CAARS‐O:L** (mean ± SD)					
*Inattention*	73.73 ± 9.88	72.02 ± 10.86	0.665	0.508	0.165
*Hyperactivity*	52.81 ± 12.96	62.32 ± 10.00	−3.244	**0.002**	0.821
*Impulsiveness*	59.62 ± 11.83	64.72 ± 12.61	−1.694	0.095	0.418
*Self‐concept problems*	64.77 ± 8.87	60.55 ± 12.23	1.545	0.127	0.395
*ADHD Global Index*	67.85 ± 10.87	69.47 ± 11.32	−0.594	0.554	0.146
**CGI‐S** (mean ± SD)	4.63 ± 0.49	4.88 ± 0.43	−2.271	**0.027**	0.532
**FAST** (mean ± SD)	29.87 ± 8.74	30.34 ± 10.91	−0.202	0.841	0.048
**WHODAS 2.0** (mean ± SD)	23.59 ± 11.84	27.61 ± 17.88	−1.172	0.245	0.265
**BDI‐II** (mean ± SD)	14.31 ± 8.65	16.67 ± 9.20	−1.120	0.266	0.265
**STAI state** (mean ± SD)	66.17 ± 25.83	71.43 ± 24.72	−0.893	0.375	0.208

**Abbreviations:** ADHD‐RS, Attention Deficit/Hyperactivity Disorder Rating Scale; BDI‐II, Beck Depression Inventory‐II; CAARS, Conners' Adult ADHD Rating Scales; CGI‐S, Clinical Global Impression‐Severity; FAST, Functioning Assessment Short Test; SD, Standard deviation; STAI, State–Trait Anxiety Inventory; WHODAS 2.0, World Health Organization Disability Assessment Schedule 2.0; WURS, Wender Utah Rating Scale.

Clinically, the combined group presented with greater severity across several domains. This group retrospectively reported higher childhood ADHD symptoms (WURS; *t* = −2.143, *p* = 0.035), greater current core symptom severity (ADHD‐RS; *t* = −4.614, *p* < 0.001) and higher clinician‐rated severity (CGI‐S; *t* = −2.271, *p* = 0.027). Self‐reported CAARS‐S:L scores mirrored this pattern, with the combined group scoring higher on inattention (*t* = −2.042, *p* = 0.045), hyperactivity (*t* = −6.105, *p* < 0.001), impulsiveness (*t* = −2.478, *p* = 0.015) and the ADHD global index (*t* = −3.768, *p* < 0.001). Observer ratings (CAARS‐O:L) revealed a between‐group difference only for hyperactivity (*t* = −3.244, *p* = 0.002). No significant differences were found for depressive symptoms (BDI‐II), anxiety (STAI) or functional impairment (FAST, WHODAS). After Bonferroni correction (adjusted *α* = 0.002), significant differences were retained only for the ADHD‐RS total score, self‐reported CAARS hyperactivity and global index, as well as observer‐rated CAARS hyperactivity.

### 3.2 Efficacy of Intervention by ADHD Presentations and CBT Format (Pre–Post Analysis)

Table 3 presents the differential treatment response by ADHD presentation. Significant time × presentation interactions emerged for core symptom severity (ADHD‐RS; *F* = 10.995, *p* = 0.001, *ηp* ^2^ = 0.135) and self‐reported hyperactivity (CAARS‐S:L; *F* = 4.471, *p* = 0.038, *ηp* ^2^ = 0.065), indicating a steeper trajectory of improvement in the combined group relative to the inattentive group, with moderate‐to‐large and moderate effect sizes, respectively. No time × presentation × format interactions were detected at this stage.

**TABLE 3:** The efficacy of CBT intervention across ADHD predominant presentations was assessed through pre‐ and posttreatment evaluations (*N* = 80).

Scales	Time	12‐Session		6‐Session		Time			Time x treatment duration			Time x presentation			Time x treatment x presentation		
		Inattentive	Combined	Inattentive	Combined	*F*	*p*	*ηp* ^2^	*F*	*p*	*ηp* ^2^	*F*	*p*	*ηp* ^2^	*F*	*p*	*ηp* ^2^
**ADHD‐RS**	Pre	30.846 (1.613)	35.667 (1.119)	31.059 (1.410)	38.609 (1.213)	297.251	**< 0.001**	0.808	0.325	0.571	0.005	10.995	**0.001**	0.135	0.542	0.584	0.015
	Post	19.756 (1.665)	20.808 (1.154)	21.862 (1.445)	23.518 (1.283)												
**CAARS‐S:L**																	
*Inattention*	Pre	72.382 (2.764)	72.038 (1.890)	67.353 (2.337)	76.870 (2.009)	57.202	**< 0.001**	0.453	0.007	0.935	0.000	0.653	0.422	0.009	2.439	0.094	0.063
	Post	62.447 (2.868)	61.990 (1.989)	59.631 (2.463)	65.037 (2.229)												
*Hyperactivity*	Pre	53.824 (2.561)	63.654 (1.764)	49.588 (2.181)	65.348 (1.875)	25.543	**< 0.001**	0.285	0.114	0.736	0.002	4.471	**0.038**	0.065	0.980	0.380	0.028
	Post	49.857 (2.637)	56.638 (1.836)	47.568 (2.273)	57.764 (2.036)												
*Impulsiveness*	Pre	58.841 (3.492)	65.808 (2.403)	59.353 (2.972)	67.913 (2.555)	19.317	**< 0.001**	0.230	0.567	0.454	0.009	1.147	0.288	0.017	0.755	0.474	0.021
	Post	56.555 (3.598)	57.797 (2.505)	52.242 (3.100)	60.471 (2.780)												
*Self‐concept problems*	Pre	58.837 (2.946)	63.308 (2.018)	60.882 (2.496)	64.391 (2.146)	10.337	**0.002**	0.131	0.031	0.861	0.000	0.737	0.394	0.011	0.228	0.797	0.006
	Post	56.804 (3.051)	57.226 (2.119)	56.623 (2.623)	59.598 (2.368)												
*ADHD Global Index*	Pre	65.564 (2.635)	72.077 (1.802)	64.176 (2.228)	74.043 (1.916)	36.684	**< 0.001**	0.361	0.042	0.838	0.001	1.368	0.247	0.021	0.354	0.703	0.010
	Post	59.565 (2.735)	63.460 (1.897)	57.968 (2.349)	64.611 (2.126)												
**CAARS‐O:L**																	
*Inattention*	Pre	75.377 (2.816)	71.600 (1.889)	72.255 (2.401)	72.500 (2.013)	66.663	**< 0.001**	0.514	0.804	0.373	0.013	1.136	0.291	0.018	0.382	0.684	0.011
	Post	64.897 (2.816)	64.566 (2.017)	60.492 (2.401)	62.427 (2.173)												
*Hyperactivity*	Pre	56.986 (3.022)	63.120 (2.022)	49.637 (2.579)	61.409 (2.155)	12.886	**0.001**	0.176	0.058	0.810	0.001	0.196	0.659	0.003	1.025	0.365	0.031
	Post	50.556 (3.022)	59.121 (2.170)	47.499 (2.579)	54.429 (2.338)												
*Impulsiveness*	Pre	57.797 (3.189)	63.920 (2.150)	60.463 (2.712)	65.636 (2.292)	15.179	**< 0.001**	0.197	2.117	0.151	0.033	0.646	0.425	0.010	0.017	0.983	0.001
	Post	55.957 (3.189)	59.873 (2.272)	54.845 (2.712)	58.348 (2.442)												
*Self‐concept problems*	Pre	61.887 (2.997)	59.440 (2.018)	65.906 (2.550)	61.818 (2.151)	25.338	**< 0.001**	0.294	1.268	0.265	0.020	3.582	0.063	0.056	0.077	0.926	0.002
	Post	55.473 (2.997)	56.681 (2.138)	56.060 (2.550)	57.204 (2.300)												
*ADHD Global Index*	Pre	66.182 (2.833)	68.440 (1.900)	68.746 (2.415)	70.636 (2.025)	37.269	**< 0.001**	0.381	6.553	**0.013**	0.098	0.049	0.826	0.001	0.238	0.789	0.007
	Post	62.923 (2.833)	63.109 (2.029)	57.749 (2.415)	60.640 (2.185)												
**CGI‐S**	Pre	4.462 (0.176)	4.852 (0.122)	4.765 (0.154)	4.913 (0.132)	280.556	**< 0.001**	0.796	0.101	0.751	0.001	1.176	0.282	0.016	0.512	0.601	0.014
	Post	3.184 (0.182)	3.229 (0.126)	3.381 (0.158)	3.505 (0.141)												
**FAST**	Pre	30.077 (2.603)	30.074 (1.806)	29.706 (2.277)	30.652 (1.957)	140.601	**< 0.001**	0.671	3.225	0.077	0.045	1.609	0.209	0.023	0.037	0.964	0.001
	Post	21.085 (2.643)	19.097 (1.833)	23.210 (2.303)	22.431 (2.011)												
**WHODAS 2.0**	Pre	27.083 (4.104)	27.800 (2.843)	21.118 (3.448)	27.381 (3.102)	29.071	**< 0.001**	0.311	0.007	0.932	0.000	0.014	0.905	0.000	0.392	0.677	0.012
	Post	17.990 (4.378)	18.485 (2.927)	12.496 (3.521)	18.171 (3.213)												
**BDI‐II**	Pre	14.468 (2.240)	16.577 (1.547)	14.059 (1.913)	16.783 (1.645)	48.707	**< 0.001**	0.412	0.574	0.451	0.008	0.272	0.604	0.004	0.017	0.983	0.000
	Post	9.677 (2.300)	11.160 (1.584)	8.254 (1.947)	9.893 (1.714)												
**STAI State**	Pre	60.556 (7.379)	58.120 (5.169)	57.333 (6.673)	56.714 (5.640)	5.981	**0.017**	0.091	0.001	0.970	0.000	0.000	0.999	0.000	0.498	0.610	0.015
	Post	55.070 (7.621)	47.281 (5.509)	46.750 (7.244)	51.468 (6.237)												

*Note:* Data are presented as estimated marginal means (standard error). *F* and *p* values are derived from Type III tests of fixed effects in linear mixed models. Effect sizes are reported as partial eta‐squared (*ηp* ^2^). Statistically significant values (*p* < 0.05) are highlighted in bold.

Abbreviations: ADHD‐RS, Attention Deficit/Hyperactivity Disorder Rating Scale; BDI‐II, Beck Depression Inventory‐II; CAARS, Conners' Adult ADHD Rating Scales; CGI‐S, Clinical Global Impression‐Severity; FAST, Functioning Assessment Short Test; STAI, State–Trait Anxiety Inventory; WHODAS 2.0, World Health Organization Disability Assessment Schedule 2.

Beyond presentation‐specific effects, the intervention produced robust, generalized clinical gains. A significant main effect of time reflected pre–post improvements across the full sample, with large effect sizes for core symptoms (ADHD‐RS; *F* = 297.251, *p* < 0.001, *ηp* ^2^ = 0.808), clinician‐rated severity (CGI‐S; *F* = 280.556, *p* < 0.001, *ηp* ^2^ = 0.796) and functional impairment (FAST: *F* = 140.601, *p* < 0.001, *ηp* ^2^ = 0.671; WHODAS: *F* = 29.071, *p* < 0.001, *ηp* ^2^ = 0.311). Comparable large effects were observed across all CAARS symptom domains, depression (BDI‐II; *F* = 48.707, *p* < 0.001, *ηp* ^2^ = 0.412) and state anxiety (STAI‐State; *F* = 5.981, *p* = 0.017, *ηp* ^2^ = 0.091). With respect to treatment format, a time × format interaction was found only for the observer‐rated CAARS ADHD Global Index (*F* = 6.553, *p* = 0.013, *ηp* ^2^ = 0.098), favouring the six‐session format, with no corresponding effect on self‐reports.

### 3.3 Clinical Significance: Reliable Change Index

Reliable improvement was achieved by 94.5% of participants on core ADHD symptoms (ADHD‐RS) and 98.6% on clinician‐rated severity (CGI‐S). Rates were lower for functional outcomes—35.6% on the FAST and 41.2% on the WHODAS 2.0—consistent with the greater inertia typically observed in functional recovery. Reliable improvement on CAARS subscales ranged from 16.7% to 42.4% for self‐reported measures and from 14.5% to 43.5% for observer‐rated measures. Reliable improvement in state anxiety (STAI‐State) was observed in only 7.2% of participants. Detailed RCI classifications are provided in Supplementary Table S1.

### 3.4 Longitudinal Effects at 3‐ and 6‐Month Follow‐Ups

Sixty‐eight percent of participants (*n* = 55; 21 inattentive, 34 combined) completed the 6‐month follow‐up. Attrition was nondifferential; no baseline demographic or clinical differences were observed between completers and noncompleters (Supplementary Table S2).

**FIGURE 1:**
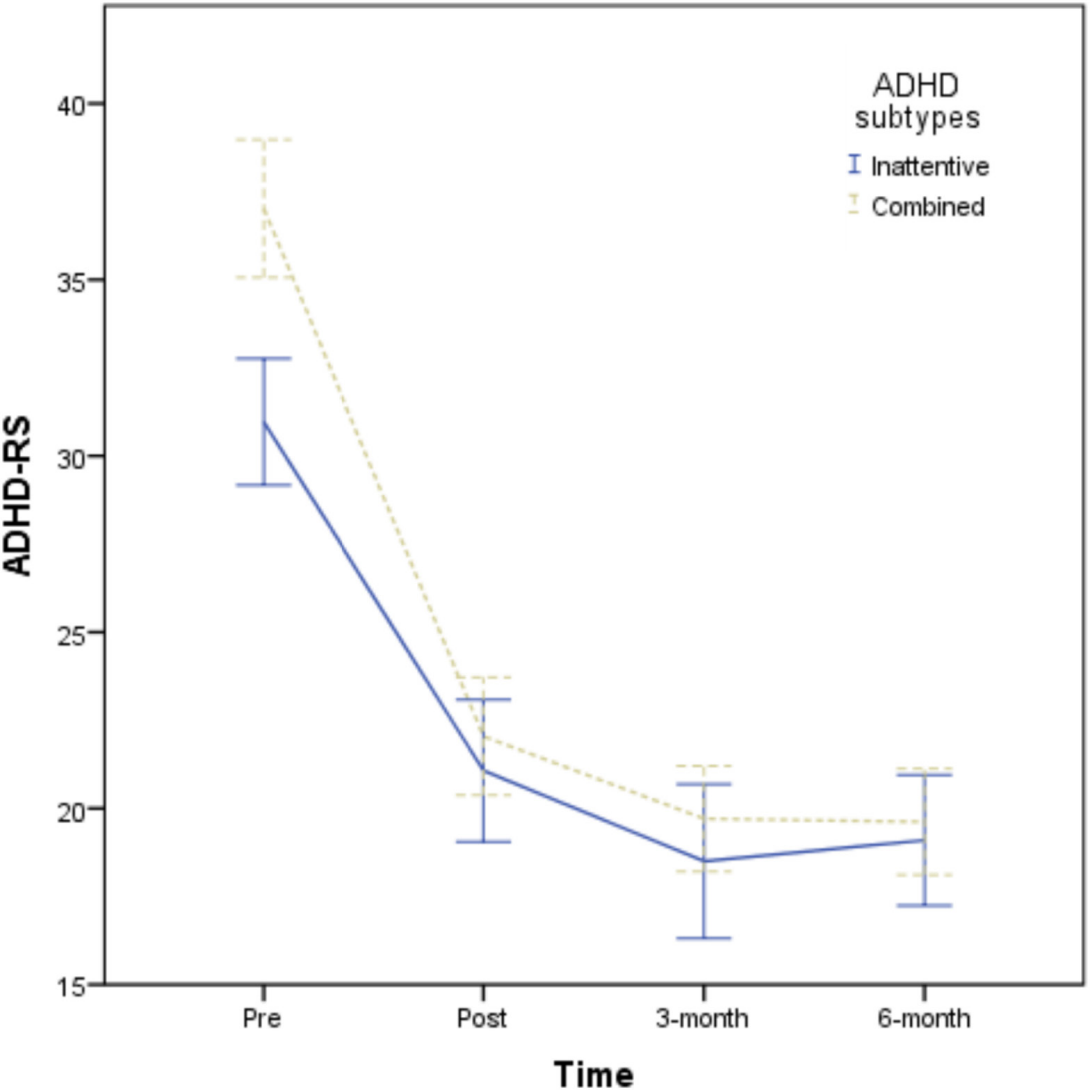
Change in ADHD symptom severity (ADHD‐RS) across baseline, posttreatment and follow‐up assessments (3 and 6 months), by ADHD predominant presentation.

Longitudinal analyses (Table 4) confirmed the durability of the differential presentation response. The time × presentation interaction persisted for core ADHD symptoms (ADHD‐RS; *F* = 7.741, *p* < 0.001, *ηp* ^2^ = 0.111) and self‐reported CAARS hyperactivity (*F* = 5.458, *p* = 0.001, *ηp* ^2^ = 0.092), representing moderate‐to‐large and moderate effect sizes, respectively, with the combined group maintaining a steeper trajectory of improvement throughout follow‐up. The symptomatic evolution of the primary outcome (ADHD‐RS) is illustrated in Figure 1.

**TABLE 4:** Efficacy of CBT intervention across predominant presentations was also evaluated at 3‐ and 6‐month follow‐up assessments (*n* = 55).

Scales	Time	12‐Session		6‐Session		Time			Time x treatment duration			Time x presentation			Time x treatment x presentation		
		Inattentive	Combined	Inattentive	Combined	*F*	*p*	*ηp* ^2^	*F*	*p*	*ηp* ^2^	*F*	*p*	*ηp* ^2^	*F*	*p*	*ηp* ^2^
**ADHD‐RS**	Pre	30.846 (1.477)	35.667 (1.025)	31.059 (1.292)	38.609 (1.111)	286.406	**< 0.001**	0.822	1.497	0.217	0.024	7.741	**< 0.001**	0.111	0.406	0.804	0.012
	Post	19.758 (1.517)	20.761 (1.051)	21.799 (1.318)	23.554 (1.163)												
	3 m	16.227 (1.555)	18.338 (1.064)	19.906 (1.342)	22.367 (1.221)												
	6 m	18.135 (1.647)	18.960 (1.136)	19.354 (1.430)	21.003 (1.267)												
**CAARS‐S:L**																	
*Inattention*	Pre	72.284 (2.575)	72.038 (1.778)	67.353 (2.199)	76.870 (1.890)	52.035	**< 0.001**	0.483	0.068	0.977	0.001	0.764	0.516	0.014	1.373	0.247	0.041
	Post	62.489 (2.665)	62.003 (1.855)	59.415 (2.296)	64.756 (2.060)												
	3 m	62.671 (2.665)	60.911 (1.880)	58.032 (2.401)	63.671 (2.187)												
	6 m	60.554 (2.940)	58.980 (2.168)	57.261 (2.608)	61.957 (2.289)												
*Hyperactivity*	Pre	53.768 (2.424)	63.654 (1.686)	49.588 (2.085)	65.348 (1.792)	19.443	**< 0.001**	0.264	0.229	0.876	0.004	5.458	**0.001**	0.092	0.627	0.644	0.020
	Post	49.784 (2.489)	56.494 (1.739)	47.460 (2.152)	57.804 (1.911)												
	3 m	50.512 (2.489)	55.736 (1.756)	48.213 (2.222)	57.556 (1.996)												
	6 m	50.711 (2.671)	55.870 (1.948)	48.101 (2.360)	55.347 (2.065)												
*Impulsiveness*	Pre	58.967 (3.274)	65.808 (2.277)	59.353 (2.816)	67.913 (2.421)	19.633	**< 0.001**	0.267	0.330	0.804	0.006	0.900	0.442	0.016	1.456	0.220	0.045
	Post	56.456 (3.360)	57.801 (2.348)	51.907 (2.905)	60.634 (2.578)												
	3 m	55.092 (3.360)	56.718 (2.370)	50.859 (2.999)	60.394 (2.692)												
	6 m	56.041 (3.601)	54.099 (2.625)	50.618 (3.182)	60.316 (2.784)												
*Self‐concept problems*	Pre	58.683 (2.721)	63.308 (1.876)	60.882 (2.321)	64.391 (1.995)	9.350	**< 0.001**	0.143	0.213	0.887	0.004	0.962	0.412	0.017	0.391	0.815	0.012
	Post	56.674 (2.818)	57.159 (1.960)	56.510 (2.427)	59.566 (2.181)												
	3 m	56.401 (2.818)	56.079 (1.988)	55.566 (2.543)	58.935 (2.319)												
	6 m	57.277 (3.121)	55.275 (2.305)	55.052 (2.770)	56.685 (2.432)												
*ADHD Global Index*	Pre	65.546 (2.667)	72.077 (1.837)	64.176 (2.272)	74.043 (1.953)	30.063	**< 0.001**	0.352	0.385	0.764	0.007	2.308	0.078	0.040	0.170	0.953	0.005
	Post	59.425 (2.765)	63.397 (1.922)	57.708 (2.380)	64.536 (2.142)												
	3 m	58.879 (2.765)	61.687 (1.951)	56.303 (2.498)	60.553 (2.284)												
	6 m	59.469 (3.076)	60.413 (2.276)	56.719 (2.732)	59.547 (2.399)												
**CAARS‐O:L**																	
*Inattention*	Pre	75.446 (2.555)	71.600 (1.723)	72.103 (2.176)	72.500 (1.837)	51.405	**< 0.001**	0.495	0.368	0.776	0.007	0.979	0.404	0.018	0.319	0.865	0.010
	Post	64.912 (2.565)	64.583 (1.834)	60.539 (2.185)	62.361 (1.974)												
	3 m	63.367 (2.565)	62.942 (1.895)	59.753 (2.294)	62.860 (2.099)												
	6 m	63.522 (2.836)	59.981 (2.212)	58.800 (2.499)	59.234 (2.200)												
*Hyperactivity*	Pre	56.877 (2.760)	63.120 (1.866)	49.559 (2.348)	61.409 (1.990)	8.607	**< 0.001**	0.147	0.513	0.674	0.010	0.810	0.490	0.016	0.786	0.537	0.027
	Post	50.812 (2.771)	59.081 (1.976)	47.584 (2.358)	54.593 (2.126)												
	3 m	51.448 (2.771)	57.871 (2.036)	49.272 (2.464)	56.263 (2.247)												
	6 m	51.938 (3.033)	57.589 (2.344)	48.357 (2.664)	53.578 (2.345)												
*Impulsiveness*	Pre	57.586 (2.975)	63.920 (2.021)	60.346 (2.526)	65.636 (2.155)	9.508	**< 0.001**	0.156	2.254	0.084	0.042	1.730	0.163	0.032	0.096	0.984	0.003
	Post	56.097 (2.987)	59.912 (2.120)	54.893 (2.537)	58.296 (2.278)												
	3 m	56.915 (2.987)	60.556 (2.173)	55.922 (2.631)	57.603 (2.385)												
	6 m	58.158 (3.217)	58.405 (2.448)	54.518 (2.808)	55.503 (2.472)												
*Self‐concept problems*	Pre	61.588 (2.773)	59.440 (1.885)	65.756 (2.353)	61.818 (2.009)	17.622	**< 0.001**	0.256	1.769	0.155	0.033	2.413	0.069	0.045	0.562	0.691	0.019
	Post	55.649 (2.783)	56.740 (1.975)	56.095 (2.363)	57.163 (2.120)												
	3 m	57.558 (2.783)	56.447 (2.022)	54.644 (2.449)	57.234 (2.218)												
	6 m	55.670 (2.991)	56.452 (2.270)	54.645 (2.608)	57.655 (2.296)												
*ADHD Global Index*	Pre	66.120 (2.563)	68.440 (1.730)	68.731 (2.182)	70.636 (1.844)	27.656	**< 0.001**	0.352	4.926	**0.003**	0.088	0.176	0.912	0.003	0.323	0.862	0.011
	Post	63.054 (2.573)	63.155 (1.838)	57.745 (2.191)	60.572 (1.978)												
	3 m	62.327 (2.573)	63.525 (1.897)	57.399 (2.296)	60.137 (2.098)												
	6 m	62.817 (2.834)	61.300 (2.203)	55.968 (2.494)	58.566 (2.195)												
**CGI‐S**	Pre	4.462 (0.198)	4.852 (0.138)	4.765 (0.174)	4.913 (0.149)	209.363	**< 0.001**	0.768	1.148	0.331	0.018	0.486	0.692	0.008	0.306	0.873	0.009
	Post	3.192 (0.204)	3.224 (0.141)	3.383 (0.177)	3.507 (0.157)												
	3 m	2.733 (0.210)	2.914 (0.143)	3.139 (0.181)	3.318 (0.165)												
	6 m	2.488 (0.223)	2.759 (0.154)	3.005 (0.194)	3.170 (0.172)												
**FAST**	Pre	30.077 (2.411)	30.074 (1.673)	29.706 (2.108)	30.652 (1.812)	145.833	**< 0.001**	0.705	3.940	**0.009**	0.061	1.156	0.328	0.019	0.042	0.997	0.001
	Post	21.073 (2.446)	19.087 (1.696)	23.218 (2.131)	22.432 (1.859)												
	3 m	16.425 (2.478)	16.751 (1.706)	19.789 (2.152)	20.747 (1.909)												
	6 m	14.615 (2.555)	14.753 (1.767)	18.539 (2.225)	20.151 (1.948)												
**WHODAS** **2.0**	Pre	27.083 (3.532)	27.800 (2.447)	21.118 (2.968)	27.381 (2.670)	36.807	**< 0.001**	0.398	0.683	0.564	0.012	0.166	0.919	0.003	0.295	0.881	0.010
	Post	17.839 (3.749)	18.464 (2.519)	12.496 (3.030)	18.177 (2.764)												
	3 m	12.288 (3.640)	14.213 (2.553)	11.149 (3.090)	15.506 (2.976)												
	6 m	11.946 (4.014)	10.908 (2.814)	9.583 (3.398)	13.375 (3.041)												
**BDI‐II**	Pre	14.450 (1.960)	16.577 (1.353)	14.059 (1.674)	16.783 (1.439)	40.831	**< 0.001**	0.404	0.506	0.679	0.008	0.245	0.865	0.004	0.222	0.926	0.007
	Post	9.706 (2.028)	11.151 (1.391)	8.283 (1.708)	9.841 (1.510)												
	3 m	8.433 (2.028)	8.771 (1.409)	5.706 (1.742)	8.426 (1.589)												
	6 m	8.531 (2.235)	8.426 (1.543)	5.333 (1.913)	7.454 (1.651)												
**STAI State**	Pre	64.388 (6.785)	66.654 (4.693)	68.647 (5.804)	76.826 (4.990)	19.056	**< 0.001**	0.241	0.808	0.491	0.013	0.060	0.981	0.001	2.522	**0.044**	0.070
	Post	57.936 (7.006)	50.732 (4.871)	47.838 (5.917)	63.449 (5.219)												
	3 m	57.664 (7.006)	47.891 (4.871)	42.619 (6.024)	61.709 (5.472)												
	6 m	53.823 (7.669)	46.148 (5.303)	41.238 (6.569)	62.071 (5.671)												

*Note:* Data are presented as estimated marginal means (standard error). *F* and *p* values are derived from Type III tests of fixed effects in linear mixed models. Effect sizes are reported as partial eta‐squared (*ηp* ^2^). Statistically significant values (*p* < 0.05) are highlighted in bold.

**Abbreviations:** ADHD‐RS, Attention Deficit/Hyperactivity Disorder Rating Scale; BDI‐II, Beck Depression Inventory‐II; CAARS, Conners' Adult ADHD Rating Scales; CGI‐S, Clinical Global Impression‐Severity; FAST, Functioning Assessment Short Test; STAI, State–Trait Anxiety Inventory; WHODAS 2.0, World Health Organization Disability Assessment Schedule 2.0.

A three‐way interaction (time × presentation × format) emerged for state anxiety (STAI‐State; *F* = 2.522, *p* = 0.044, *ηp* ^2^ = 0.070), suggesting that long‐term anxiety outcomes depend on the interplay between treatment length and clinical presentation. No other three‐way interactions were identified.

The broad clinical gains observed at posttreatment were maintained through the 6‐month follow‐up. Significant main effects of time (all *p* < 0.001) were sustained with large effect sizes for core symptoms (ADHD‐RS; *F* = 286.406, *ηp* ^2^ = 0.822), clinician‐rated severity (CGI‐S: *F* = 209.363, *ηp* ^2^ = 0.768) and functional outcomes (FAST: *F* = 145.833, *ηp* ^2^ = 0.705; WHODAS 2.0: *F* = 36.807, *ηp* ^2^ = 0.398). Treatment duration shaped long‐term trajectories in two specific domains: The 12‐session format produced greater functional gains over time (FAST time × format: *F* = 3.940, *p* = 0.009, *ηp* ^2^ = 0.061), while the six‐session format maintained its advantage on the observer‐rated CAARS Global Index (*F* = 4.926, *p* = 0.003, *ηp* ^2^ = 0.088), both with moderate effect sizes.

### 3.5 Sensitivity Analyses

Complete‐case analyses restricted to 6‐month completers corroborated the primary intention‐to‐treat results, with significant time main effects retained for the ADHD‐RS, FAST and WHODAS 2.0. When baseline severity was included as a subject‐level covariate, the time × presentation interaction for the ADHD‐RS remained significant (*p* = 0.001), confirming a genuine differential treatment response rather than a statistical artefact. In contrast, the interaction for self‐reported CAARS hyperactivity became nonsignificant, indicating that initial severity differences largely accounted for the divergent trajectories in this domain. Consistent with unadjusted models, interactions for the CGI‐S and observer‐rated CAARS hyperactivity remained nonsignificant.

## 4 Discussion

The present study demonstrates that CBT produces significant improvements in core ADHD symptoms, psychosocial functioning and comorbid emotional symptoms in adults, with gains maintained regardless of treatment format and clinical presentation. The practical significance of these effects is underscored by RCI analyses: 94.5% of participants achieved reliable improvement in core symptoms (ADHD‐RS) and 98.6% in clinician‐rated severity (CGI‐S). These findings reinforce the clinical utility of both the standard and abbreviated CBT formats (Corrales et al. 2024) and align with a broad body of evidence supporting CBT's positive impact in this population (Yang et al. 2025; Philipsen et al. 2015; Gonda et al. 2024).

At baseline, participants with the combined presentation showed greater symptom severity than those with the inattentive presentation. After applying the Bonferroni correction (adjusted *α* = 0.002), significant differences were confined to core ADHD symptom severity (ADHD‐RS) and two CAARS‐S:L subscales—hyperactivity and the ADHD global index—with no significant differences in functioning, depression or anxiety. This pattern suggests that presentation differences at baseline primarily reflect core ADHD symptomatology rather than a broader clinical burden and is consistent with reports of higher symptom severity in the combined presentation (Mowlem et al. 2019; Wu et al. 2022). However, findings across the literature remain inconsistent (Molavi et al. 2020), possibly due to differences in sample characteristics and the use of corrections for multiple comparisons.

The pre–post analysis confirmed robust improvements across all clinical and functional domains (Corrales et al. 2024; Philipsen et al. 2015; Knouse and Safren 2010), with large effect sizes for time (*ηp* ^2^ ranging from 0.311 to 0.822) indicating gains of substantial clinical magnitude. While meta‐analyses have established CBT's efficacy for core ADHD symptoms (Tourjman et al. 2022), samples in those reviews often exclude individuals with significant comorbidities. The present findings extend this evidence, demonstrating improvements not only in ADHD symptoms but also in anxiety and depression—albeit with more modest reliable change rates for comorbid outcomes (e.g., 7.2% for state anxiety), consistent with the known resilience of these domains to short‐term intervention.

No main effects of treatment format were observed for most outcomes, with the exception of the observer‐rated CAARS Global Index. Significant time × presentation interactions emerged for core symptom severity (ADHD‐RS) and self‐reported hyperactivity, with the combined group showing a steeper trajectory of improvement. However, interpretation requires caution: while the ADHD‐RS interaction was statistically robust (*p* = 0.001), the hyperactivity interaction reached only nominal significance and did not survive Bonferroni adjustment. Crucially, the time × presentation interaction for the ADHD‐RS survived adjustment for baseline severity as a covariate, confirming that the differential trajectory reflects a genuine treatment effect rather than regression to the mean.

Sustained improvements were observed across all clinical and functional domains through the 6‐month follow‐up. While some evidence suggests a decline in CBT benefits beyond the initial months (Li and Zhang 2023), our results align with research showing that gains can persist for at least 12 months (López‐Pinar et al. 2018), suggesting successful internalization of compensatory strategies into daily functioning. The few format‐specific interactions observed—notably, the 12‐session format producing greater long‐term functional gains (FAST; *ηp* ^2^ = 0.061)—likely reflect the additional time available for consolidating complex skills. Nonetheless, the absence of three‐way interactions indicates that both formats were broadly effective across ADHD presentations, supporting the exploration of more personalized and flexible delivery models.

Several limitations warrant consideration. First, the inconsistency between the stringent Bonferroni correction applied at baseline (*p* ≤ 0.002) and the nominal significance threshold used in the mixed‐effects models (*p* < 0.05) means that interactions not meeting the conservative criterion should be regarded as exploratory. Second, the absence of a waitlist or active control group limits causal inference and precludes ruling out spontaneous remission or practice effects; however, the RCI rates observed (94%–98%) are difficult to attribute to these factors alone. Additional limitations include reliance on self‐report data, differential attrition at follow‐up and the absence of sex‐disaggregated analyses, all of which restrict the generalizability of the findings. Future studies should incorporate objective neuropsychological measures of attention and executive functioning to better characterize presentation‐specific treatment mechanisms and guide the development of personalized interventions.

## 5 Conclusions

This study provides preliminary evidence that structured CBT effectively improves ADHD symptoms, psychosocial functioning and emotional distress in adults, with approximately 95% of participants achieving reliable symptomatic improvement. Although individuals with the combined presentation showed a more pronounced reduction in core symptoms, these differential trajectories did not consistently survive conservative corrections for multiple comparisons and should therefore be interpreted as a promising but preliminary finding rather than a definitive clinical rule. Importantly, the absence of significant three‐way interactions indicates that CBT was broadly effective across presentations and treatment formats, with gains maintained for at least 6 months. Future randomized controlled trials with active control conditions are needed to confirm these presentation‐specific patterns and better control for the effects of regression to the mean.

## Author Contributions

**Juan Jesús Crespín:** formal analysis, data curation, investigation, writing – review and editing. **Montse Corrales:** conceptualization, investigation, data curation, methodology, writing – original draft. **Vanesa Richarte:** methodology, data curation, investigation, writing – review and editing. **Gemma Parramon‐Puig:** investigation, writing – review and editing. **Paula Querol:** investigation, data curation, writing – review and editing. Ferran Mestres: investigation, writing – review and editing. **Carolina Ramos‐Sayalero:** data curation, writing – review and editing. **Maria Boix:** data curation, writing – review and editing. **Carla Torrent:** formal analysis, methodology, writing – review and editing. **Christian Fadeuilhe:** methodology, data curation, investigation, writing – review and editing. **Josep Antoni Ramos‐Quiroga:** conceptualization, supervision, methodology, writing – review and editing, project administration, funding acquisition. **Silvia Amoretti:** conceptualization, supervision, methodology, writing—original draft, writing – review and editing.

## Funding

The authors received no specific funding for this work.

## Ethics Statement

The study was approved by the Clinical Research Ethics Committee of the Vall d'Hebron University Hospital. The study participants have provided written informed consent for their participation in the study.

This work constitutes a secondary analysis derived from a randomized controlled trial (RCT) conducted at the Adult ADHD Program of the Hospital Universitari Vall d'Hebron (Barcelona, Spain), as described by Corrales et al. (2024).

Ethical approval was obtained from the hospital's Clinical Research Ethics Committee, and the study was registered on ClinicalTrials.gov (NCT04588181).

## Conflicts of Interest

Dr. Vanesa Richarte declares that she has given lectures or received help to attend conferences from Rubió and Shire/Takeda. Dr. Christian Fadeuilhe declares that he has given lectures or received help to attend conferences from Rubió and Shire/Takeda. Dr. Montse Corrales declares that she has received help to attend conferences from Shire/Takeda. Dr. Silvia Amoretti has been a consultant to and/or has received honoraria/grants from Otsuka‐Lundbeck, with no financial or other relationship relevant to the subject of this article. Professor Josep Antoni Ramos‐Quiroga was on the speakers' bureau and/or acted as consultant for Biogen, Idorsia, Casen‐Recordati, Johnson&Johnson, Novartis, Takeda, Bial, Sincrolab, Neuraxpharm, Novartis, BMS, Medice, Rubió, Uriach, Technofarma and Raffo in the last 3 years. He also received travel awards (air tickets + hotel) for taking part in psychiatric meetings from Idorsia, Johnson&Johnson, Rubió, Takeda, Bial and Medice. The Department of Psychiatry chaired by him received unrestricted educational and research support from the following companies in the last 3 years: Exeltis, Idorsia, Casen‐Recordati, Takeda, Neuraxpharm, Oryzon, Roche, Probitas and Rubió, as well as Johnson&Johnson.

The other authors declare no conflicts of interest.

## Supporting information


**Table S1:** Proportion of participants showing clinically meaningful change according to the Reliable Change Index (RCI).
**Table S2:** Comparison of baseline characteristics between participants who completed the 6‐month follow‐up and those who did not.

## Data Availability

The data that support the findings of this study are available on request from the corresponding authors.
