# Supplementary material for: Does Attention‐Deficit/Hyperactivity Disorder Predominant Presentation Matter? Examining Functional and Symptom Changes After Cognitive Behavioural Therapy
Source: Clin Psychol Psychother. 2026 Apr 17;33(2):e70271. doi: 10.1002/cpp.70271 (PMC13088220; doi:10.1002/cpp.70271)
Supplement: Supplementary file 1 — Table S1: Proportion of participants showing clinically meaningful change according to the Reliable Change Index (RCI). Table S2: Comparison of baseline characteristics between participants who completed the 6‐month follow‐up and those who did not. [file CPP-33-e70271-s001.docx]

**Supplementary Table 1.** Proportion of participants showing clinically meaningful change according to the Reliable Change Index (RCI).

| **Outcome** | **Reliable improvement (%)** | **No reliable change (%)** | **Reliable deterioration (%)** |
| --- | --- | --- | --- |
| **ADHD-RS** | 94.5 | 5.5 | 0.0 |
| **CAARS-S:L** | | | |
| *Inattention* | 42.4 | 57.6 | 0.0 |
| *Hyperactivity* | 16.7 | 83.3 | 0.0 |
| *Impulsiveness* | 21.2 | 75.8 | 3.0 |
| *Self-concept problems* | 18.2 | 75.8 | 6.1 |
| ADHD Global Index | 28.8 | 71.2 | 0.0 |
| **CAARS-O:L** | | | |
| *Inattention* | 43.5 | 56.5 | 0.0 |
| *Hyperactivity* | 14.5 | 82.3 | 3.2 |
| *Impulsiveness* | 14.5 | 82.3 | 3.2 |
| *Self-concept problems* | 16.1 | 79.0 | 4.8 |
| *ADHD Global Index* | 32.3 | 66.1 | 1.6 |
| **CGI-S** | 98.6 | 1.4 | 0.0 |
| **FAST** | 35.6 | 64.4 | 0.0 |
| **WHODAS 2.0** | 41.2 | 54.4 | 4.4 |
| **BDI-II** | 30.0 | 68.6 | 1.4 |
| **STAI state** | 7.2 | 91.3 | 1.4 |

**Note**: Participants were classified as showing reliable improvement (RCI ≤ −1.96), no reliable change (−1.96 < RCI < 1.96), or reliable deterioration (RCI ≥ 1.96).

**Abbreviations**: ADHD-RS: Attention Deficit/Hyperactivity Disorder Rating Scale; BDI-II: Beck Depression Inventory-II; CAARS: Conners' Adult ADHD Rating Scales; CGI-S: Clinical Global Impression-Severity; FAST: Functioning Assessment Short Test; STAI: State-Trait Anxiety Inventory; WHODAS 2.0: World Health Organization Disability Assessment Schedule 2.0.

**Supplementary Table 2.** Comparison of baseline characteristics between participants who completed the 6-month follow-up and those who did not.

|  | **Completers (n=55)** | **Non-completers (n=25)** | **t** | **p or χ²** | **Effect size** |
| --- | --- | --- | --- | --- | --- |
| **Age** (mean±SD) | 42.80±7.98 | 37.88±11.19 | -1.981 | 0.055 | 0.506 |
| **Sex** (*Males*, %) | 28 (50.90) | 18 (72.0) | 3.129 | 0.077 | 0.198 |
| **Educational level**: university (n, %) | 18 (32.70) | 5 (20.00) | 2.489 | 0.477 | 0.176 |
| **Marital status**: single (n, %) | 13 (23.60) | 12 (48.00) | 5.420 | 0.067 | 0.26 |
| **Living arrangements**: alone (n, %) | 5 (9.10) | 3 (12.00) | 6.398 | 0.269 | 0.283 |
| **Employment status**: Employed (n, %) | 29 (52.70) | 10 (40.00) | 3.883 | 0.566 | 0.220 |
| **WURS** (mean±SD) | 53.38±15.78 | 53.58±15.04 | 0.052 | 0.959 | 0.013 |
| **ADHD-RS** (mean±SD) | 34.42±6.22 | 35.48±8.10 | 0.642 | 0.523 | 0.147 |
| **CAARS-S:L** (mean±SD) | | | | |  |
| *Inattention* | 71.87±10.24 | 73.96±10.45 | 0.826 | 0.412 | 0.202 |
| *Hyperactivity* | 59.43±10.34 | 59.96±12.83 | 0.195 | 0.846 | 0.045 |
| *Impulsiveness* | 64.20±12.26 | 63.42±16.39 | -0.235 | 0.815 | 0.053 |
| *Self-concept problems* | 61.87±12.10 | 63.88±9.15 | 0.724 | 0.471 | 0.187 |
| ADHD Global Index | 70.00±9.49 | 69.79±11.59 | -0.083 | 0.934 | 0.019 |
| **CAARS-O:L** (mean±SD) | | | | |  |
| *Inattention* | 72.84±10.63 | 72.14±10.36 | -0.263 | 0.794 | 0.066 |
| *Hyperactivity* | 58.24±10.93 | 60.55±14.25 | 0.754 | 0.453 | 0.181 |
| *Impulsiveness* | 64.27±12.60 | 59.73±11.92 | -1.437 | 0.155 | 0.370 |
| *Self-concept problems* | 60.80±12.16 | 64.95±8.41 | 1.455 | 0.150 | 0.396 |
| *ADHD Global Index* | 69.25±10.77 | 68.05±12.08 | -0.424 | 0.673 | 0.104 |
| **CGI-S** (mean±SD) | 4.80±0.45 | 4.76±0.52 | -0.352 | 0.726 | 0.082 |
| **FAST** (mean±SD) | 30.31±10.13 | 29.84±10.21 | -0.192 | 0.849 | 0.046 |
| **WHODAS 2.0** (mean±SD) | 26.45±16.19 | 25.09±15.34 | -0.337 | 0.737 | 0.086 |
| **BDI-II** (mean±SD) | 15.83±9.78 | 15.71±7.18 | -0.056 | 0.955 | 0.014 |
| **STAI state** (mean±SD) | 68.43±26.42 | 71.83±22.17 | 0.551 | 0.583 | 0.139 |

**Abbreviations:** ADHD-RS: Attention Deficit/Hyperactivity Disorder Rating Scale; BDI-II: Beck Depression Inventory-II;CAARS: Conners’ Adult ADHD Rating Scales; CGI-S: Clinical Global Impression-Severity; FAST: Functioning Assessment Short Test; SD: Standard deviation; STAI: State-Trait Anxiety Inventory; WHODAS 2.0: World Health Organization Disability Assessment Schedule 2.0; WURS: Wender Utah Rating Scale.
